# Supplementary material for: Computational Modelling for Electrical Impedance Spectroscopy-Based Diagnosis of Oral Potential Malignant Disorders (OPMD)
Source: Sensors (Basel). 2022 Aug 8;22(15):5913. doi: 10.3390/s22155913 (PMC9371412; doi:10.3390/s22155913)
Supplement: Supplementary file 1 [file sensors-22-05913-s001.zip › sensors-1842873-supplementary.pdf]

Computational Modelling for Electrical Impedance Spectroscopy-based Diagnosis of Oral Potential Malignant Disorders (OPMD)

Supplementary material

S1 Cellular –level morphological data:

S1.1 Histology-derived cell dimensions

Table S1. A list of average cell dimensions measured from histology images. Note colour coding refers to diagnosis.

				Mean cell dimensions/µm					
**Case**	**Diagnosis**	**Tissue type**	**oral site**	Basal XY	Basal Z	Prickle XY	Prickle Z	Superficial XY	Superficial Z
1	normal	Non-keratinous	Buccal mucosa	6.10	11.01	26.85	14.80	32.87	15.84
2	normal	Keratinous	Hard palate	7.85	15.68	19.88	9.83	33.00	5.90
3	Severe dysplasia	Para keratinous	ventral tongue	11.08	22.10	33.45	15.71	41.56	13.90
4	Severe dysplasia	Para keratinous	lateral tongue	12.81	25.44	27.88	13.77	37.08	10.51
5	Moderate dysplasia	Para keratinous	lateral tongue	8.26	15.59	23.94	11.55	28.49	8.71
6	Hyperkeratosis, no dysplasia	Keratinous	Hard palate	10.11	15.06	19.78	13.71	26.91	10.43
7	Severe dysplasia	Para - keratinous	lateral tongue	12.58	27.98	28.04	14.68	50.54	9.58

S1.2 Assumed Extracellular space dimensions

Table S2. List of extracellular space thickness used for oral tissue cellular derived from values for cervical cell types

Pathology	Cell (oral tissue)	Cell (cervix)	ECS thickness/μm
normal	Basal	Basal	0.4
Moderate dysplasia	Basal	Basal	0.6
Severe dysplasia	Basal	Basal	0.8
normal	Prickle	intermediate	0.1
Moderate dysplasia	Prickle	intermediate	0.4
Severe dysplasia	Prickle	intermediate	0.6
normal	Superficial	Superficial	0.05
Moderate dysplasia	Superficial	Superficial	0.2
Severe dysplasia	Superficial	Superficial	0.4

Note: No literature values for expansion of ECS in oral tissue due to dysplasia exist. Equivalence with ECS thickness expansion in cervical cancer (Walker, 2001, Walker et al, 2003) was assumed for similar cell types. Here it is assumed that cervical cancer grade CINII and CINIII are equivalent to moderate and severe dysplasia in oral tissue respectively. Which cells are deemed equivalent and ECS thickness values that are used for various degrees of dysplasia severity are listed in Table S2.

S1.3 Cellular electrical material properties

Table S3. List of material properties and thickness of cellular model components.

Component	Conductivity/Sm^{-1}	Relative permittivity	Thickness/μm	References
ECS	1.1	72	0.05-0.8 (pathology/cell type dependant)	(Irimajiri et al., 1978, Walker et al., 2002)
Cytoplasm	0.6	86	(depends on cell size)	(Irimajiri et al., 1978, Asami et al., 1989)
Nucleus	0.8	145	4 (diameter)	(Irimajiri et al., 1978, Bordi et al., 1990, Asami et al., 1989)
Cell membrane	1e-7	10	0.008	(Irimajiri et al., 1978, Bordi et al., 1993, Asami et al., 1989, Huang et al., 1995)
Nuclear membrane	2e-3	10	0.04	(Irimajiri et al., 1978)

S2 Additional Finite element Methodology details

To find the value of electric potential throughout the model, Maxwell's equations must be solved, including a capacitive and resistive component of tissue. From Maxwell's continuity equation:

$$-\nabla.(\nabla\phi(\sigma + j\omega\varepsilon_0\varepsilon_r)) = -\nabla.\boldsymbol{j} \quad \text{(S1)}$$

Where ϕ is the electric potential and $\boldsymbol{j}$ is the current density. As charge is conserved, the current flowing into the system is equal to the current flowing out of the system and hence equation A1 is equal to zero. From $\boldsymbol{j}$ we can calculate current flowing into the tissue and hence impedance from equation 1. For equation A1 to hold it is assumed that there is no induction, material properties are time invariant and isotropic (at the cellular scale). The model solves the electric potential in each node (each point on an element) for an arbitrary geometry for (Dirichlet) boundary conditions such as an applied voltage or current. ANSYS also implicitly applies a Neumann boundary condition to all free surfaces (outside of the model with no applied current or voltage), setting the current density to zero, this confines current flow within the model geometry. As the applied AC voltage or current is an ideal sinusoid, a harmonic analysis is used, greatly reducing the computation time.

In this study both prism (also referred to as wedges) and tetrahedron elements are used. Tetrahedrons perform better for regions with significant volume (e.g. the cytoplasm in the cellular scale or the stroma at the tissue scale). Conversely, where very thin regions are required (cell membranes and the thinner layers in the tissue scale model), prism elements perform better. For this project the electric solid element types employed are listed in table S4. The current flowing through the cellular scale model is found using the ANSYS function "RFOR".

Table S4. List of element types used in this project.

Element type	Description	Purpose
SOLID231	20 node, hexahedral	Can be transformed (degenerated) into prisms or tetrahedrons.
SOLID232	10 node, tetrahedral	Convert degenerate SOLID231 to SOLID231 to reduce memory usage (less nodes).
PLANE230	8-node square	Can be degenerated into triangle elements (useful for initial surface mesh of tissue scale model electrodes).

S3 Model convergence studies

S3.1 Cellular level mesh convergence study

The impedance spectrum of a single cell model with dimensions of 15µm by 15µm by 15µm was simulated for the frequency range 76Hz to 10MHz for models with increasingly fine mesh. Fig. S1A shows the low frequency real impedance shows adequate convergence at ~0.6 million degrees of freedom. This translated as a minimum mesh size of 0.81 µm in the nucleus and 0.71 µm in the

cytoplasm. Membranes were meshed using extruded prisms (two layers per membrane) from the tetrahedral meshes in the direction of nucleus to outwards. This mesh size was used for all cellular scale models. Fig. S1B shows the spectra of meshes with 0.19, 0.64 and 1.28 million degrees of freedom, not there is no visible difference with respect to the whole spectra.

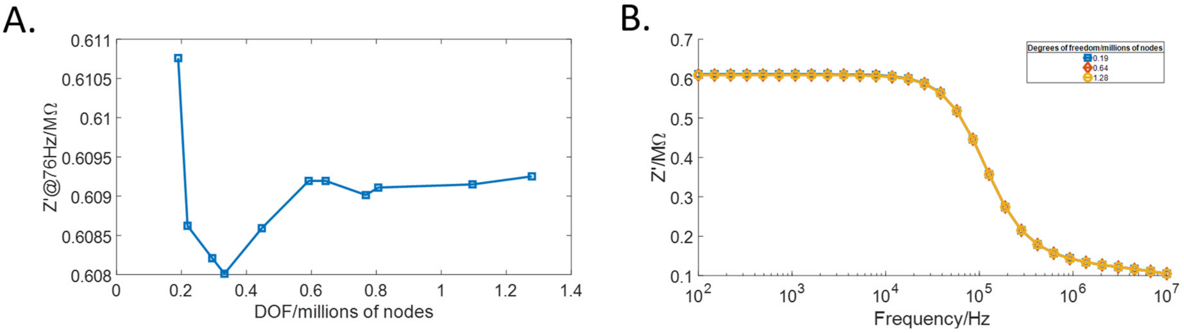

Figure S1. Mesh convergence study for the cellular scale model. (A) Low frequency impedance value versus degrees of freedom. (B) Spectra for several different meshes sizes.

S3.2 Tissue scale model mesh convergence study.

The impedance spectrum of a tissue scale model (epithelial -keratinous model) with dimensions of 40 mm by 40 mm by 5 mm was simulated for the frequency range 76Hz to 10MHz for models with increasingly fine mesh. Fig S2 shows the low frequency real impedance shows sharp convergence at ~0.25 million degrees of freedom. As a factor of safety models with ~0.85 million degrees of freedom were used. This translated as a minimum mesh size of 1.4mm. This was decreased to 0.04 mm for the electrode areas. This mesh size was used for all tissue scale models.

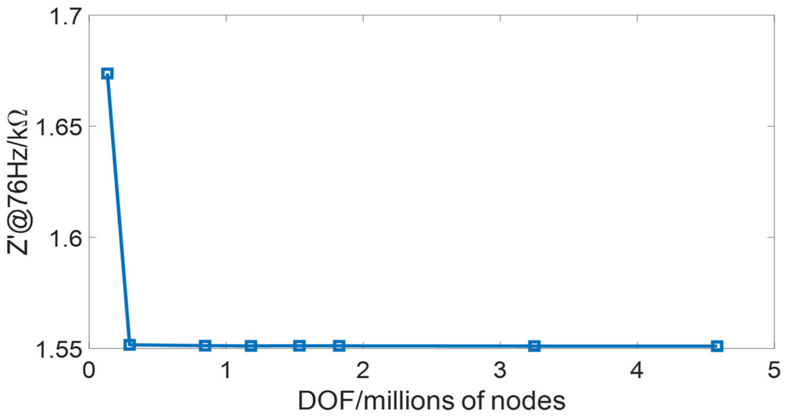

Figure S2. Convergence study for the tissue scale model. Impedance at low frequency versus degrees of freedom.

S3.3 Cellular scale size effects

The required number of repeating symmetry units in the XY and Z direction (see fig. 4, main text) was obtained by increasing the number of repeating units until convergence was achieved. For the XY direction adding additional symmetry units had negligible effect (see fig. S3A). For the Z direction, acceptable convergence was found with five repeating units (see fig. S3B). For all cellular simulations

in the XY direction one repeating unit was used. For all Z direction cellular simulations five repeating units were used.

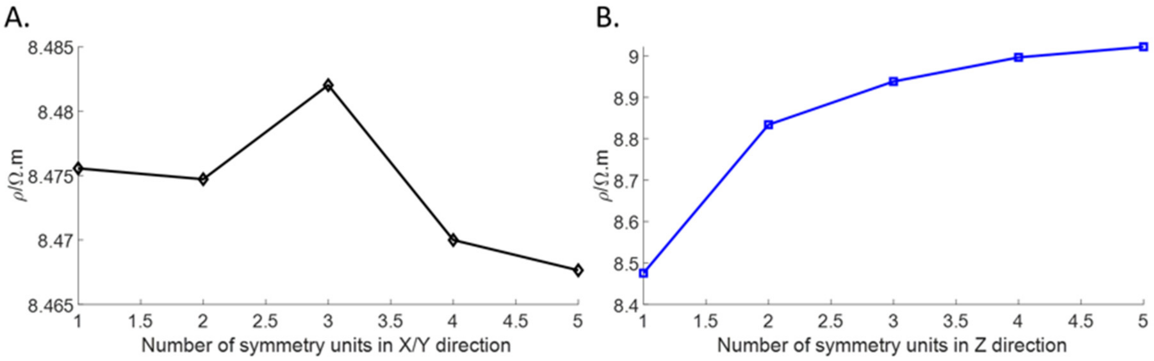

Figure S3. Impedivity versus number of repeating units in the XY (A) and Z (B) directions.

S3.4 Tissue scale size effects

To find the correct stroma depth for the tissue scale model (epithelial -keratinous model) a healthy (keratinous) and cancerous (severe dysplasia, non-keratinous) model was simulated. Note, although the cancerous tissue was from a non-keratinous site, the pathological keratinisation made the two tissues comparable in terms of keratinous layer thickness and hence the main difference was the cellular layer properties. The depth of the stroma was increased from 0.3 mm to 5 mm. For the cancerous tissue there was a significant change in the spectra from 0.3 mm to 1 mm (see fig. S4A). For the healthy spectra there was little observable change (see fig. S4B). Comparing the low frequency impedance values, convergence was more gradual for the cancerous example (see fig. S4C) than the normal example (see fig. S4D). As a catch all the stroma depth was set to always make up the tissue depth of the model 5 mm including the thickness of other tissue layers.

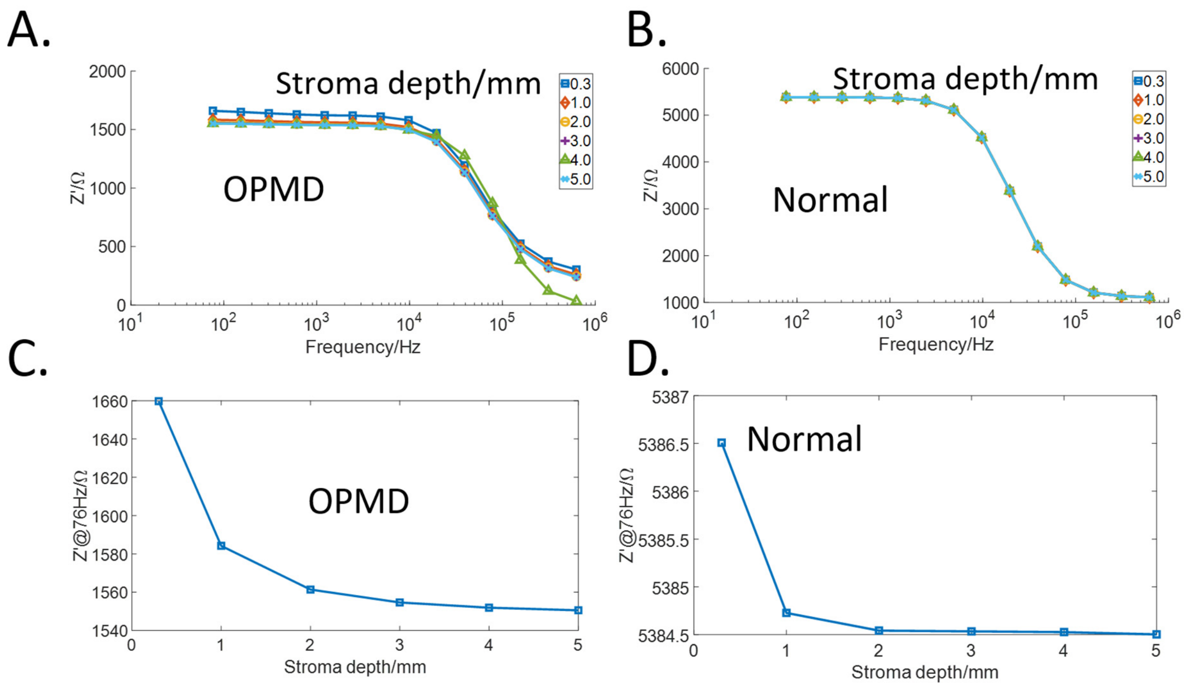

Figure S4. Spectra of (A) OPMD and (B) normal oral tissue with increasing stroma depth. Low frequency impedance of (C) OPMD and (D) normal oral tissue with increasing stroma depth.

S4 Cellular scale simulation results.

Cellular scale models that were optimised to remove size effects and had a mesh size dictated by a convergence study (see previous sections) were simulated for basal, prickle and superficial cells of seven histology images. A list of all cellular scale dimensions is given in Table S1 and extracellular space dimensions in table S2. The impedivity of each cell type was simulated in the Z and XY directions. It should be noted that whilst the cell types are listed by tissue type (e.g. degree of keratinisation) no keratinous layer is present at the cellular scale. Any difference in impedivity is resultant from dysplasia (i.e. differences in cell morphology and ECS).

The impedivity of basal cells in the Z direction showed little trend with pathology (see table S5). In contrast the Z direction impedivity of prickle and superficial cells showed an increase in real impedivity for healthy cells (see table S5). It is noted that the cells with moderate dysplasia have similar impedivities to those with severe dysplasia but the highest value for all dysplastic cells, in good agreement with pathology.

Cellular simulations were repeated in the XY direction for basal, prickle and superficial cells (see table S6). In contrast to the Z direction results, the XY cellular scale simulations did not show any trend with pathology and had lower impedivity values at low frequency. Imaginary impedivity, used to calculate permittivity for the tissue scale simulations is given in tables S7 and S8 for the Z and XY directions respectively.

Table S5. Cellular scale Z direction real impedivity spectra.

		Real Impedivity/Ω.m						
Cell	frequency/Hz	Case: 1	2	3	4	5	6	7
Basal	76.29	18.61	23.67	16.94	19.47	16.84	15.54	19.09
Basal	152.59	18.61	23.67	16.94	19.47	16.84	15.54	19.09
Basal	305.18	18.61	23.67	16.94	19.47	16.84	15.54	19.08
Basal	610.35	18.61	23.67	16.94	19.47	16.84	15.54	19.08
Basal	1220.70	18.61	23.67	16.94	19.47	16.84	15.54	19.08
Basal	2441.41	18.61	23.66	16.94	19.46	16.84	15.54	19.06
Basal	4882.81	18.61	23.62	16.93	19.44	16.84	15.54	18.98
Basal	9765.63	18.60	23.47	16.89	19.36	16.82	15.53	18.70
Basal	19531.30	18.55	22.93	16.73	19.04	16.75	15.49	17.87
Basal	39062.50	18.36	21.46	16.15	17.94	16.47	15.34	16.09
Basal	78125.00	17.69	18.52	14.43	15.14	15.51	14.80	13.18
Basal	156250.00	15.70	14.23	11.47	11.46	13.15	13.21	10.29
Basal	312500.00	12.26	10.77	9.14	9.21	10.23	10.57	8.72
Basal	625000.00	9.55	9.09	8.05	8.19	8.52	8.55	7.95
Prickle	76.29	337.89	254.43	71.26	59.09	76.50	59.17	59.17
Prickle	152.59	337.87	254.43	71.26	59.09	76.50	59.17	59.17
Prickle	305.18	337.77	254.41	71.26	59.09	76.50	59.17	59.17
Prickle	610.35	337.41	254.35	71.25	59.08	76.50	59.17	59.17
Prickle	1220.70	335.96	254.09	71.25	59.08	76.49	59.16	59.16
Prickle	2441.41	330.42	253.08	71.22	59.07	76.47	59.15	59.15
Prickle	4882.81	311.24	249.16	71.10	59.01	76.39	59.08	59.08
Prickle	9765.63	259.70	235.48	70.62	58.80	76.06	58.84	58.84
Prickle	19531.30	167.40	198.03	68.78	57.97	74.79	57.87	57.87
Prickle	39062.50	77.12	129.54	62.40	54.91	70.15	54.34	54.34
Prickle	78125.00	32.36	61.00	46.42	45.71	56.70	44.18	44.18
Prickle	156250.00	17.21	26.47	25.99	29.30	34.40	27.49	27.49
Prickle	312500.00	11.97	14.81	14.18	16.08	17.82	15.21	15.21
Prickle	625000.00	9.69	10.88	10.00	10.57	11.25	10.32	10.32
Superficial	76.29	822.08	843.76	138.21	125.58	192.14	87.80	180.48
Superficial	152.59	821.57	843.72	138.21	125.58	192.14	87.80	180.48
Superficial	305.18	819.58	843.57	138.21	125.58	192.14	87.80	180.48
Superficial	610.35	811.77	842.98	138.19	125.58	192.12	87.80	180.47
Superficial	1220.70	782.84	840.64	138.12	125.56	192.05	87.79	180.42
Superficial	2441.41	693.33	831.44	137.86	125.49	191.78	87.77	180.26
Superficial	4882.81	505.77	797.37	136.83	125.23	190.72	87.68	179.61
Superficial	9765.63	270.62	691.14	132.94	124.17	186.64	87.30	177.04
Superficial	19531.30	108.85	467.12	120.38	120.14	172.73	85.84	167.57
Superficial	39062.50	43.24	218.88	91.03	106.55	136.93	80.50	138.83
Superficial	78125.00	21.63	80.69	50.82	74.77	80.53	64.97	85.77
Superficial	156250.00	13.77	30.49	23.60	37.91	36.03	38.97	39.43
Superficial	312500.00	10.39	15.37	13.09	17.94	17.15	19.42	17.97
Superficial	625000.00	9.04	10.84	9.77	11.09	11.15	11.65	10.94

Table S6. Cellular scale XY direction real impedivity spectra.

		Real Impedivity/Ω.m						
Cell	frequency/Hz	Case: 1	2	3	4	5	6	7
Basal	76.29	4.51	5.97	4.23	4.87	4.13	3.51	4.94
Basal	152.59	4.51	5.97	4.23	4.87	4.13	3.51	4.94
Basal	305.18	4.51	5.97	4.23	4.87	4.13	3.51	4.94
Basal	610.35	4.51	5.97	4.23	4.87	4.13	3.51	4.94
Basal	1220.70	4.51	5.97	4.23	4.87	4.13	3.51	4.94
Basal	2441.41	4.51	5.97	4.23	4.87	4.13	3.51	4.94
Basal	4882.81	4.51	5.97	4.23	4.87	4.13	3.51	4.94
Basal	9765.63	4.51	5.97	4.23	4.87	4.13	3.51	4.94
Basal	19531.30	4.51	5.96	4.22	4.86	4.13	3.51	4.92
Basal	39062.50	4.50	5.93	4.20	4.81	4.12	3.50	4.88
Basal	78125.00	4.46	5.79	4.11	4.63	4.07	3.45	4.70
Basal	156250.00	4.33	5.32	3.81	4.07	3.90	3.29	4.14
Basal	312500.00	3.90	4.18	3.08	3.02	3.39	2.85	3.08
Basal	625000.00	3.01	2.79	2.21	2.12	2.53	2.18	2.14
Prickle	76.29	4.50	29.08	8.13	7.06	8.89	7.37	7.37
Prickle	152.59	4.50	29.08	8.13	7.06	8.89	7.37	7.37
Prickle	305.18	4.50	29.04	8.13	7.06	8.89	7.37	7.37
Prickle	610.35	4.50	28.92	8.12	7.06	8.89	7.37	7.37
Prickle	1220.70	4.50	28.42	8.12	7.05	8.88	7.37	7.36
Prickle	2441.41	4.50	26.63	8.11	7.05	8.87	7.36	7.36
Prickle	4882.81	4.49	21.52	8.05	7.02	8.84	7.33	7.33
Prickle	9765.63	4.47	13.30	7.84	6.93	8.70	7.23	7.23
Prickle	19531.30	4.40	7.32	7.13	6.59	8.19	6.86	6.86
Prickle	39062.50	4.13	4.73	5.45	5.60	6.75	5.78	5.78
Prickle	78125.00	3.43	3.38	3.49	3.90	4.47	3.97	3.97
Prickle	156250.00	2.49	2.53	2.41	2.59	2.85	2.62	2.62
Prickle	312500.00	1.91	2.06	1.98	2.03	2.17	2.04	2.04
Prickle	625000.00	1.66	1.82	1.74	1.77	1.86	1.78	1.78
Superficial	76.29	12.14	5.70	11.59	9.29	14.83	8.56	8.89
Superficial	152.59	12.14	5.70	11.59	9.29	14.82	8.56	8.89
Superficial	305.18	12.14	5.70	11.57	9.29	14.81	8.56	8.87
Superficial	610.35	12.14	5.70	11.52	9.29	14.75	8.56	8.79
Superficial	1220.70	12.13	5.70	11.31	9.28	14.52	8.56	8.51
Superficial	2441.41	12.09	5.68	10.57	9.24	13.70	8.55	7.60
Superficial	4882.81	11.91	5.63	8.57	9.08	11.33	8.50	5.69
Superficial	9765.63	11.28	5.44	5.61	8.52	7.46	8.31	3.79
Superficial	19531.30	9.42	4.84	3.64	6.96	4.59	7.66	2.83
Superficial	39062.50	6.18	3.65	2.77	4.60	3.32	6.01	2.36
Superficial	78125.00	3.64	2.58	2.27	2.99	2.62	3.87	2.03
Superficial	156250.00	2.55	2.08	1.94	2.31	2.16	2.61	1.81
Superficial	312500.00	2.08	1.83	1.75	1.96	1.88	2.09	1.68
Superficial	625000.00	1.81	1.67	1.64	1.75	1.72	1.82	1.60

Table S7. Cellular scale Z direction imaginary impedivity spectra.

		Imaginary Impedivity/Ω.m						
Cell	frequency/Hz	Case: 1	2	3	4	5	6	7
Basal	76.29	3.21E-03	1.19E-02	5.53E-03	8.78E-03	3.71E-03	2.54E-03	1.42E-02
Basal	152.59	6.42E-03	2.39E-02	1.11E-02	1.76E-02	7.42E-03	5.07E-03	2.84E-02
Basal	305.18	1.28E-02	4.77E-02	2.21E-02	3.51E-02	1.48E-02	1.01E-02	5.69E-02
Basal	610.35	2.57E-02	9.54E-02	4.42E-02	7.02E-02	2.97E-02	2.03E-02	1.14E-01
Basal	1220.70	5.13E-02	1.91E-01	8.85E-02	1.40E-01	5.93E-02	4.06E-02	2.27E-01
Basal	2441.41	1.03E-01	3.81E-01	1.77E-01	2.81E-01	1.19E-01	8.12E-02	4.53E-01
Basal	4882.81	2.05E-01	7.58E-01	3.53E-01	5.60E-01	2.37E-01	1.62E-01	8.94E-01
Basal	9765.63	4.10E-01	1.49E+00	7.04E-01	1.11E+00	4.73E-01	3.24E-01	1.70E+00
Basal	19531.30	8.16E-01	2.78E+00	1.38E+00	2.16E+00	9.39E-01	6.45E-01	2.94E+00
Basal	39062.50	1.60E+00	4.63E+00	2.59E+00	3.88E+00	1.82E+00	1.27E+00	4.33E+00
Basal	78125.00	3.00E+00	6.45E+00	4.11E+00	5.54E+00	3.24E+00	2.36E+00	5.08E+00
Basal	156250.00	4.77E+00	6.84E+00	4.54E+00	5.23E+00	4.50E+00	3.70E+00	4.24E+00
Basal	312500.00	5.28E+00	5.18E+00	3.41E+00	3.61E+00	4.14E+00	4.02E+00	2.84E+00
Basal	625000.00	3.97E+00	3.39E+00	2.25E+00	2.36E+00	2.84E+00	3.00E+00	1.91E+00
Prickle	76.29	1.45E+00	5.29E-01	4.96E-02	2.97E-02	4.25E-02	3.21E-02	3.21E-02
Prickle	152.59	2.91E+00	1.06E+00	9.93E-02	5.93E-02	8.51E-02	6.42E-02	6.42E-02
Prickle	305.18	5.81E+00	2.12E+00	1.99E-01	1.19E-01	1.70E-01	1.28E-01	1.28E-01
Prickle	610.35	1.16E+01	4.23E+00	3.97E-01	2.37E-01	3.40E-01	2.57E-01	2.57E-01
Prickle	1220.70	2.31E+01	8.44E+00	7.94E-01	4.74E-01	6.80E-01	5.13E-01	5.13E-01
Prickle	2441.41	4.50E+01	1.68E+01	1.59E+00	9.49E-01	1.36E+00	1.03E+00	1.03E+00
Prickle	4882.81	8.28E+01	3.28E+01	3.17E+00	1.90E+00	2.72E+00	2.05E+00	2.05E+00
Prickle	9765.63	1.30E+02	6.07E+01	6.29E+00	3.77E+00	5.41E+00	4.08E+00	4.08E+00
Prickle	19531.30	1.55E+02	9.66E+01	1.22E+01	7.42E+00	1.06E+01	8.00E+00	8.00E+00
Prickle	39062.50	1.25E+02	1.16E+02	2.18E+01	1.39E+01	1.97E+01	1.49E+01	1.49E+01
Prickle	78125.00	7.54E+01	9.46E+01	3.06E+01	2.23E+01	3.08E+01	2.31E+01	2.31E+01
Prickle	156250.00	4.11E+01	5.74E+01	2.80E+01	2.49E+01	3.28E+01	2.45E+01	2.45E+01
Prickle	312500.00	2.21E+01	3.12E+01	1.79E+01	1.82E+01	2.31E+01	1.72E+01	1.72E+01
Prickle	625000.00	1.19E+01	1.68E+01	9.93E+00	1.05E+01	1.32E+01	9.87E+00	9.87E+00
Superficial	76.29	1.07E+01	3.06E+00	1.99E-01	1.00E-01	2.40E-01	4.92E-02	1.89E-01
Superficial	152.59	2.14E+01	6.12E+00	3.97E-01	2.01E-01	4.80E-01	9.85E-02	3.78E-01
Superficial	305.18	4.26E+01	1.22E+01	7.94E-01	4.02E-01	9.60E-01	1.97E-01	7.57E-01
Superficial	610.35	8.42E+01	2.45E+01	1.59E+00	8.03E-01	1.92E+00	3.94E-01	1.51E+00
Superficial	1220.70	1.60E+02	4.87E+01	3.17E+00	1.61E+00	3.84E+00	7.88E-01	3.03E+00
Superficial	2441.41	2.72E+02	9.62E+01	6.33E+00	3.21E+00	7.66E+00	1.58E+00	6.05E+00
Superficial	4882.81	3.64E+02	1.83E+02	1.25E+01	6.41E+00	1.52E+01	3.15E+00	1.20E+01
Superficial	9765.63	3.49E+02	3.09E+02	2.41E+01	1.27E+01	2.96E+01	6.26E+00	2.37E+01
Superficial	19531.30	2.39E+02	3.99E+02	4.22E+01	2.45E+01	5.35E+01	1.23E+01	4.46E+01
Superficial	39062.50	1.36E+02	3.45E+02	5.95E+01	4.29E+01	8.03E+01	2.29E+01	7.24E+01
Superficial	78125.00	7.29E+01	2.18E+02	5.83E+01	5.75E+01	8.59E+01	3.58E+01	8.36E+01
Superficial	156250.00	3.89E+01	1.19E+02	3.95E+01	5.01E+01	6.22E+01	3.84E+01	6.39E+01
Superficial	312500.00	2.05E+01	6.17E+01	2.21E+01	3.12E+01	3.58E+01	2.71E+01	3.77E+01
Superficial	625000.00	1.07E+01	3.18E+01	1.18E+01	1.69E+01	1.90E+01	1.54E+01	2.01E+01

Table S8. Cellular scale XY direction imaginary impedivity spectra.

		Imaginary Impedivity/Ω.m						
Cell	frequency/Hz	Case: 1	2	3	4	5	6	7
Basal	76.29	3.70E-04	8.94E-04	5.71E-04	9.20E-04	4.06E-04	3.47E-04	9.20E-04
Basal	152.59	7.41E-04	1.79E-03	1.14E-03	1.84E-03	8.12E-04	6.95E-04	1.84E-03
Basal	305.18	1.48E-03	3.57E-03	2.29E-03	3.68E-03	1.62E-03	1.39E-03	3.68E-03
Basal	610.35	2.96E-03	7.15E-03	4.57E-03	7.36E-03	3.25E-03	2.78E-03	7.36E-03
Basal	1220.70	5.93E-03	1.43E-02	9.14E-03	1.47E-02	6.49E-03	5.56E-03	1.47E-02
Basal	2441.41	1.19E-02	2.86E-02	1.83E-02	2.94E-02	1.30E-02	1.11E-02	2.94E-02
Basal	4882.81	2.37E-02	5.72E-02	3.66E-02	5.89E-02	2.60E-02	2.22E-02	5.89E-02
Basal	9765.63	4.74E-02	1.14E-01	7.31E-02	1.18E-01	5.19E-02	4.44E-02	1.18E-01
Basal	19531.30	9.47E-02	2.28E-01	1.46E-01	2.34E-01	1.04E-01	8.88E-02	2.34E-01
Basal	39062.50	1.89E-01	4.52E-01	2.89E-01	4.62E-01	2.06E-01	1.77E-01	4.62E-01
Basal	78125.00	3.73E-01	8.76E-01	5.59E-01	8.71E-01	4.05E-01	3.45E-01	8.74E-01
Basal	156250.00	7.12E-01	1.55E+00	9.87E-01	1.42E+00	7.55E-01	6.35E-01	1.44E+00
Basal	312500.00	1.20E+00	2.14E+00	1.35E+00	1.65E+00	1.19E+00	9.64E-01	1.69E+00
Basal	625000.00	1.49E+00	1.93E+00	1.21E+00	1.26E+00	1.29E+00	9.93E-01	1.30E+00
Prickle	76.29	2.17E-03	2.59E-01	1.06E-02	6.37E-03	9.07E-03	6.88E-03	6.88E-03
Prickle	152.59	4.34E-03	5.17E-01	2.12E-02	1.27E-02	1.81E-02	1.38E-02	1.38E-02
Prickle	305.18	8.68E-03	1.03E+00	4.23E-02	2.55E-02	3.63E-02	2.75E-02	2.75E-02
Prickle	610.35	1.74E-02	2.06E+00	8.47E-02	5.09E-02	7.25E-02	5.50E-02	5.50E-02
Prickle	1220.70	3.47E-02	4.03E+00	1.69E-01	1.02E-01	1.45E-01	1.10E-01	1.10E-01
Prickle	2441.41	6.94E-02	7.46E+00	3.38E-01	2.03E-01	2.90E-01	2.20E-01	2.20E-01
Prickle	4882.81	1.39E-01	1.15E+01	6.69E-01	4.05E-01	5.76E-01	4.37E-01	4.37E-01
Prickle	9765.63	2.75E-01	1.22E+01	1.29E+00	7.95E-01	1.13E+00	8.59E-01	8.58E-01
Prickle	19531.30	5.36E-01	8.74E+00	2.27E+00	1.48E+00	2.09E+00	1.60E+00	1.60E+00
Prickle	39062.50	9.67E-01	5.49E+00	3.08E+00	2.34E+00	3.20E+00	2.50E+00	2.50E+00
Prickle	78125.00	1.39E+00	3.52E+00	2.71E+00	2.55E+00	3.33E+00	2.69E+00	2.69E+00
Prickle	156250.00	1.33E+00	2.20E+00	1.76E+00	1.86E+00	2.35E+00	1.94E+00	1.94E+00
Prickle	312500.00	9.05E-01	1.33E+00	1.07E+00	1.14E+00	1.43E+00	1.18E+00	1.18E+00
Prickle	625000.00	5.65E-01	7.73E-01	6.60E-01	7.08E-01	8.78E-01	7.30E-01	7.30E-01
Superficial	76.29	2.40E-02	8.10E-03	1.01E-01	1.95E-02	1.21E-01	1.03E-02	1.01E-01
Superficial	152.59	4.79E-02	1.62E-02	2.01E-01	3.90E-02	2.41E-01	2.06E-02	2.02E-01
Superficial	305.18	9.58E-02	3.24E-02	4.01E-01	7.81E-02	4.81E-01	4.12E-02	4.04E-01
Superficial	610.35	1.92E-01	6.48E-02	7.98E-01	1.56E-01	9.58E-01	8.24E-02	7.98E-01
Superficial	1220.70	3.83E-01	1.30E-01	1.56E+00	3.12E-01	1.88E+00	1.65E-01	1.52E+00
Superficial	2441.41	7.62E-01	2.58E-01	2.85E+00	6.20E-01	3.49E+00	3.29E-01	2.59E+00
Superficial	4882.81	1.50E+00	5.09E-01	4.26E+00	1.21E+00	5.42E+00	6.52E-01	3.24E+00
Superficial	9765.63	2.80E+00	9.66E-01	4.28E+00	2.23E+00	5.78E+00	1.27E+00	2.67E+00
Superficial	19531.30	4.45E+00	1.60E+00	3.01E+00	3.36E+00	4.20E+00	2.27E+00	1.76E+00
Superficial	39062.50	4.90E+00	1.91E+00	1.93E+00	3.42E+00	2.70E+00	3.21E+00	1.18E+00
Superficial	78125.00	3.58E+00	1.50E+00	1.27E+00	2.39E+00	1.78E+00	2.98E+00	8.01E-01
Superficial	156250.00	2.17E+00	9.68E-01	8.21E-01	1.48E+00	1.16E+00	1.98E+00	5.29E-01
Superficial	312500.00	1.32E+00	6.32E-01	5.12E-01	9.39E-01	7.28E-01	1.21E+00	3.40E-01
Superficial	625000.00	7.96E-01	4.16E-01	3.12E-01	5.84E-01	4.44E-01	7.57E-01	2.15E-01

S5 Frequency dependent material properties for keratinous layer.

Table S9. Frequency dependent material properties for keratinous layer properties based upon stratum corneum (Johnsen, 2010). Note the same 14 frequencies listed here are used for all simulations.

frequency/Hz	resistivity/Ω.m	relative permittivity
7.63E+01	4.90E+04	3.02E+01
1.53E+02	4.20E+04	2.69E+01
3.05E+02	3.49E+04	2.40E+01
6.10E+02	2.72E+04	2.18E+01
1.22E+03	2.12E+04	1.99E+01
2.44E+03	1.63E+04	1.88E+01
4.88E+03	1.19E+04	1.79E+01
9.77E+03	7.94E+03	1.70E+01
1.95E+04	5.14E+03	1.61E+01
3.91E+04	3.31E+03	1.59E+01
7.81E+04	1.57E+03	1.47E+01
1.56E+05	4.99E+02	1.28E+01
3.13E+05	1.50E+02	9.31E+00
6.25E+05	6.51E+01	5.67E+00
